# TGF-**β**: An Important Mediator of Allergic Disease and a Molecule with Dual Activity in Cancer Development

**DOI:** 10.1155/2014/318481

**Published:** 2014-06-11

**Authors:** Belen Tirado-Rodriguez, Enrique Ortega, Patricia Segura-Medina, Sara Huerta-Yepez

**Affiliations:** ^1^Unidad de Investigación en Enfermedades Oncológicas, Hospital Infantil de México Federico Gómez, SS, Dr. Márquez No. 162, Colonia Doctores, Delegación Cuauhtémoc, 06720 México, DF, Mexico; ^2^Instituto de Investigaciones Biomédicas, Universidad Nacional Autónoma de México, Circuito Escolar, Avenida Universidad No. 3000, Delegación Coyoacán, 04510 México, DF, Mexico; ^3^Departamento de Investigación en Hiperreactividad Bronquial, Instituto Nacional de Enfermedades Respiratorias, Calzada de Tlalpan 4502, Sección XVI, 14080 México, DF, Mexico

## Abstract

The transforming growth factor-**β** (TGF-**β**) superfamily is a family of structurally related proteins that includes TGF-**β**, activins/inhibins, and bone morphogenic proteins (BMPs). Members of the TGF-**β** superfamily regulate cellular functions such as proliferation, apoptosis, differentiation, and migration and thus play key roles in organismal development. TGF-**β** is involved in several human diseases, including autoimmune disorders and vascular diseases. Activation of the TGF-**β** receptor induces phosphorylation of serine/threonine residues and triggers phosphorylation of intracellular effectors (Smads). Once activated, Smad proteins translocate to the nucleus and induce transcription of their target genes, regulating various processes and cellular functions. Recently, there has been an attempt to correlate the effect of TGF-**β** with various pathological entities such as allergic diseases and cancer, yielding a new area of research known as “allergooncology," which investigates the mechanisms by which allergic diseases may influence the progression of certain cancers. This knowledge could generate new therapeutic strategies aimed at correcting the pathologies in which TGF-**β** is involved. Here, we review recent studies that suggest an important role for TGF-**β** in both allergic disease and cancer progression.

## 1. The TGF-*β* Superfamily

The transforming growth factor-*β* (TGF-*β*) superfamily includes 33 cytokines or ubiquitous multifunctional ligands including TGF-*β*, activins (Acts), inhibins (Inhs), Nodal proteins (Noldals), bone morphogenic protein (BMP), Müllerian inhibiting substance (MIS), and differentiation factors (GDFs). These proteins have been described in a great variety of species, both vertebrate and invertebrate, and are involved in the regulation of a large number of biological functions such as proliferation, migration, inflammation, tissue repair, immune responses, cell differentiation, and apoptosis in many different cell types in adults and during development [1–5].

TGF-*β* controls extracellular matrix (ECM) production and stimulates chemotaxis of cells including fibroblasts, lymphocytes, macrophages, and neutrophils. ECM production is the most important activity of TGF-*β* in mesenchymal cells, although it also has less evident effects on the regulation of cell proliferation. The effects of TGF-*β* on the ECM are manifested at different levels, including the promotion of ECM protein expression, the inhibition of the expression of proteases capable of degrading the ECM, the stimulation of the expression of protease inhibitors (PIs), and the regulation of integrin expression and of molecules that act as receptors for several ECM components. The sum of these effects results in an increase in ECM accumulation and cell-ECM interactions. These functions are pivotal in wound repair and explain the role of TGF-*β* in diseases such as fibrosis, abnormal healing, autoimmune disease, parasitosis, asthma, and cancer [6].

## 2. TGF-*β* Isoforms

TGF-*β* cytokine family members possess 6 highly conserved cysteine residues and are encoded by 42 open reading frames in humans, 9 in flies, and 6 in worms [7]. Although the diversity of TGF-*β* ligands leads to very different cellular responses, all ligands share a common set of sequences and structural characteristics [8]. This review specifically focuses on TGF-*β*, which is ubiquitously present in many cell types including fibroblasts, endothelium, epithelium, and smooth muscle cells. TGF-*β* is released by immune cells and can be detected in wound fluids or injuries, especially during inflammation and tissue repair [9]. There are six distinct isoforms of TGF-*β* encoded by different genes, with homologies ranging from 72% to 92%. TGF-*β* isoforms are highly conserved but diverge in various amino acid regions. TGF-*β* isoforms are expressed in mammals, including humans (TGF-*β*1, TGF-*β*2, and TGF-*β*3), birds (TGF-*β*2, TGF-*β*3, and TGF-*β*4), amphibians (TGF-*β*2, TGF-*β*5), and fish such as* Sparus aurata* (TGF-*β*6) [10, 11]. All isoforms are secreted as latent inactive precursors that require activation before binding to the TGF-*β* receptor [12, 13].

In humans, three TGF-*β* isoforms are expressed, TGF-*β*1, TGF-*β*2, and TGF-*β*3. TGF-*β*1 is the most abundant isoform and was cloned from human placenta [14]. In mice, TGF-*β*1 mRNA and/or protein have been observed in cartilage, bone, and skin, suggesting an important role in the growth and differentiation of these tissues [15]. Moreover, TGF-*β*1 has also been shown to be a very potent stimulator of chemotaxis because it stimulates monocyte, lymphocyte, neutrophil, and fibroblast migration [16, 17]. A pivotal role for TGF-*β*1 has also been described in tissue repair [18].

TGF-*β*2 was first described in human glioblastoma cells and has been shown to suppress IL-2 production. Physiologically, TGF-*β*2 is expressed in neurons, astroglia, and the embryonic central nervous system cells.

The third isoform, TGF-*β*3, was identified from a cDNA library of a human rhabdomyosarcoma cell line. TGF-*β*3 shares 80% amino acid homology with TGF-*β*1 and TGF-*β*2. Studies in mice have shown that TGF-*β*3 is essential in normal palate function and in lung morphogenesis [19, 20]. The biological effects of the different TGF-*β* isoforms depend on their availability, the combination of two types of receptors, and the intracellular signaling pathways that they induce (see below) [3].

## 3. Synthesis and Activation of TGF-*β*

The mature dimeric form of TGF-*β* consists of two monomers stabilized by hydrophobic interactions and disulfide bonds. It is the mature dimeric form of TGF-*β* that initiates intracellular signaling. TGF-*β* is secreted from cells as a latent complex, which needs to be activated to release the active TGF-*β* dimers. Activation of latent TGF-*β* complexes can be induced by various factors such as extreme pH, elevated temperatures, and latency-associated peptide proteolysis or by a particular activation mechanism mediated by the binding of the TGF-*β*1 latent complex to an ECM protein called thrombospondin-1 (TSP-1) [21].

**Figure 1:**
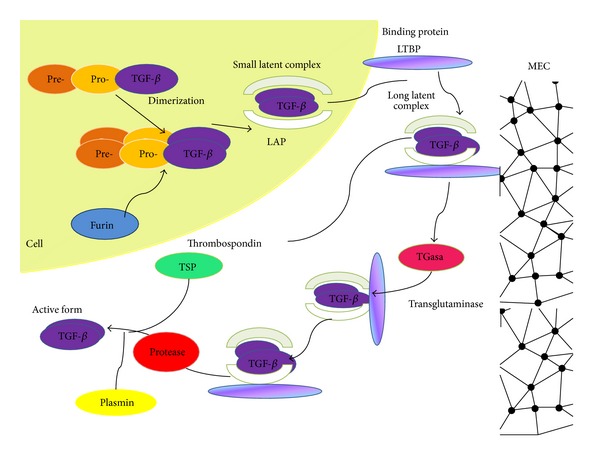
Synthesis and activation of TGF-*β*. TGF-*β* is synthesized as an inactive precursor with a preregion (signal peptide) and a proregion (N-terminal peptide LAP). Processing of the inactive form begins with the proteolytic cleavage of the signal peptide from pre-pro-TGF-*β*. After dimerization, TGF-*β* is cleaved by proteases (such as furin) at the C-terminal region in mature peptides and at the N-terminal LAP (latency-associated peptide). LAP-bound TGF-*β* forms small latent complexes (SLCs) that are transported to the extracellular matrix (ECM) where they can covalently bind to the binding protein (LTBP) to form a large latent complex released from the ECM by proteases. Then, the mature protein is cleaved from the LTBP in acidic conditions* in vitro* or by thrombospondin (TSP) or plasmin* in vivo*. Once the active TGF-*β* family member is released from the ECM, it can engage in signaling.

TGF-*β*1, TGF-*β*2, and TGF-*β*3 are synthesized as inactive precursor molecules (proproteins) composed of 390–412 amino acids (pro-TGF-*β*s). The large amino-terminal prodomains (known as latency-associated proteins, LAPs) are required for correct folding and dimerization of the carboxyl terminal domain of the growth factor (the mature peptide). Although TGF-*β*1, TGF-*β*2, and TGF-*β*3 LAPs differ significantly, TGF-*β*1 LAP may form complexes with active TGF-*β*2 and TGF-*β*3 molecules with the same or even higher affinity than with TGF-*β*1; this contributes to cross regulation of the different isoforms. After dimerization, the TGF-*β* dimer is cleaved from its propeptides in the Golgi apparatus by furin-type enzymes; however, it remains associated with its propeptides by noncovalent bonds. The dimeric TGF-*β* complex and its propeptides (LAPs) are known as the “small latent complex” (SLC). Most cell types release latent TGF-*β* into the ECM as “long latent complexes” (LLC), formed by the association of SLCs with a glycoprotein of 120–240 kDa known as latent TGF-*β* binding protein (LTBP) [22, 23] (Figure 1).

LTBP-1 binds to LAP via a disulfide bond. TGF-*β* can only be secreted by the producing cell when in a latent complex form. Several LAP mutations lead to the intracellular retention of TGF-*β*, demonstrating that TGF-*β* association with its LAP is necessary for secretion. The TGF-*β* dimer in the latent complex cannot interact with the receptors because LAP covers the binding site of the active TGF-*β* molecule to its receptors [24].

Once secreted, the latent complex may follow several paths. (1) It may be activated, which implies the separation of the active TGF-*β* dimer, which can then bind to its receptors and initiate a signaling cascade in an autocrine or paracrine manner. (2) The latent complex may enter the circulation and exert its effects in a different tissue [25]. The half-life of active TGF-*β* is only 2-3 minutes, but the half-life is 90 minutes in its latent form [24]. Usually, TGF-*β*1 is found in the plasma as the small or large latent form, and occasionally it may be found bound to other proteins such as thrombospondin, immunoglobulins (IgG), or macroglobulins [26]. (3) The latent complex may be deposited in the ECM where it generates a TGF-*β* reservoir that is subsequently liberated from the matrix according to physiological or pathophysiological requirements. Mobilization of TGF-*β* reservoirs from the ECM is another key point in the regulation of TGF-*β* activity because locally active TGF-*β* concentrations are regulated at this point [24, 27].

Several studies have reported the activation of TGF-*β* by retinoic acid and fibroblast growth factor-2 (FGF-2) in endothelial cells [28, 29] or by endotoxins and bleomycin in macrophages [30]. Moreover, a variety of molecules are involved in TGF-*β* activation. Proteases, including plasmin and matrix metalloproteinases MMP-2 and MMP-9, activate TGF-*β in vitro* [31, 32]. Other molecules that play a role in TGF-*β* activation include thrombospondin-1 [33], integrins such as *α*V*β*6 or *α*V*β*8, and reactive oxygen species (ROS) [34, 35].

## 4. Receptor Structure

Three types of cell surface receptors for TGF-*β* have been described, T*β*RI, T*β*RII, and T*β*RIII. The structural and functional properties of the receptors differ. The three receptors are expressed by most cell types [22, 24]. The three TGF-*β* isoforms bind and signal via the T*β*RI and T*β*RII receptors [22, 24].

The key structural features of the TGF-*β* receptors are three fingers in the ligand binding extracellular domain, a single transmembrane domain, and an intracellular serine-threonine kinase domain belonging to the serine/threonine kinase receptor family [8, 34, 36].

Type I TGF-*β* receptors are also known as ALKs (activin-like kinase receptors), which are characterized by a highly conserved sequence known as the GS domain, located between the transmembrane and the kinase domain and distinguished by repeated glycine and serine residues. Generally, type I receptors cannot efficiently bind their ligand if the type II receptor is absent. TGF-*β* binding to the type II receptor dimer induces the formation of a heterotetrameric complex with a type I receptor dimer via the interaction of the extracellular domains of both receptors. The type II receptor phosphorylates serine residues on the type I receptor. Ligand-dependent phosphorylation occurs in the GS domain and depends on the serine and threonine residues of the type II receptor [37]. Seven type I receptors have been described (ALK1–ALK7) [11, 37]. The type II receptor subfamily includes the TGF-*β* type II receptor (T*β*RII), the type 2 BMP receptor (BMPR-II), the antimüllerian hormone receptor (AMHR), and the type II and type III activin receptors (ActR-II and IIB). T*β*RII is constitutively phosphorylated, and upon ligand binding it phosphorylates T*β*RI [13], activating the type I receptor kinase to phosphorylate downstream intracellular effector proteins known as Smads. Smad activity regulates the transcription of genes induced by TGF-*β* [22]. Moreover, TGF-*β* ligands may interact with coreceptors such as endoglin and the betaglycan known as TBRIII (TGF-*β* type III receptor).

Betaglycan is a highly glycosylated transmembrane protein with a large extracellular domain and a short cytoplasmic tail lacking kinase activity. Betaglycan acts as a coreceptor for inhibins, promoting the interaction between inhibin and the type II receptors, ActR-II, ActR-IIB, and BMP RII [38–40].

Endoglin (CD105) is a membrane-associated dimer bound by disulfide bonds, originally identified as binding TGF-*β*1 and TGF-*β*3 [41]. Endoglin interacts with several ligands of the TGF-*β* family, including BMP2, BMP7, and activin. Endoglin is selectively expressed in cell types such as the placenta and plays a role in the development of heart hematopoietic stem cells [42, 43]. In contrast with betaglycan, endoglin ligand binding occurs independently of the association of TGF-*β* with the respective types I and II receptors [41].

## 5. Smads: Mediators of TGF-*β* Signal Transduction

Smad proteins are the main TGF-*β* signaling transducers, mediating signaling from cell surface receptors to nuclear target genes. Smad proteins possess highly conserved C-terminal and N-terminal domains known as Mad homology domains 1 and 2 (MH1 and MH2, resp.), which are connected by proline-rich regions [3]. The Smad family has 8 members, forming three subfamilies: the R-Smads (receptor regulated Smads), the Co-Smads (common Smad mediators), and the I-Smads (inhibitory Smads).

R-Smad proteins are directly activated (phosphorylated) by the type I receptor (T*β*RI) and are fundamental in establishing the specificity of the biological response. R-Smad2 and R-Smad3 are activated by TGF-*β*/activin receptors, and R-Smad1, R-Smad5, and R-Smad8 are activated by BMP receptors. A great variety of proteins have been identified that interact with the R-Smads and receptor complexes, and these proteins play key roles as chaperones for Smad recruitment and binding to their specific receptor. Among these proteins are the Smad anchor for receptor activation (SARA), the HGF-regulated tyrosine kinase substrate (Hgs), axin, embryonic liver fodrin (ELF), the TGF-*β* receptor-associated protein 1 (TRAP 1), the pseudogene of the ferritin light polypeptide (FTLP), and the Smad1 antagonistic effector (SANE) [3, 42].

Smad4 is the only Co-Smad known. It forms complexes with activated Smads so they can translocate to the nucleus. I-Smads, including Smad6, inhibit BMP signaling, and its overexpression partially inhibits TGF-*β* signaling by mimicking Smad7 activity. Smad6 competes with Smad4 for Smad1 binding, generating an inactive Smad1/6 complex. Smad7 inhibits TGF-*β*/activin signaling and if overexpressed, also it inhibits BMP signaling. The I-Smads antagonize signaling by interacting with receptor complexes and binding to type I receptors, thus preventing Smad4 phosphorylation. In the basal state, I-Smads are located in the nucleus and translocate to the cytoplasm after stimulation with TGF-*β* [42].

### 5.1. TGF-*β* Signaling Pathways

**Figure 2:**
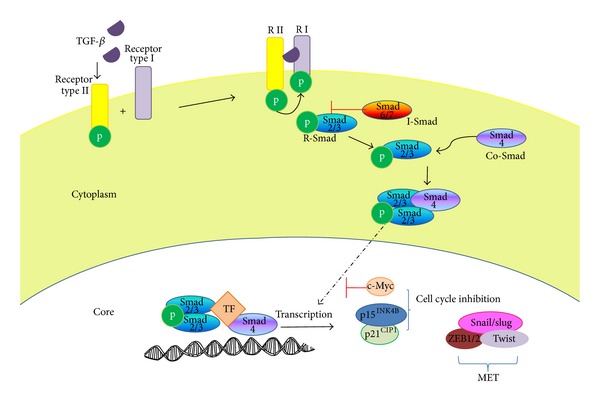
The TGF-*β* canonical signaling pathway. After the ligand binds to T*β*RII, the TGF-*β* receptors are dimerized and recruit Smad proteins. The Smad2 and/or Smad3 complex is phosphorylated by T*β*RI and forms a complex with Smad4. This complex subsequently translocates to the nucleus where it binds to specific transcription factors (TF) and induces the transcription of TGF-*β* dependent genes.

The Smad pathway is the canonical TGF-*β* signaling pathway and is directly activated by TGF-*β*. Signaling begins with recognition of the TGF-*β* ligand by T*β*RII. This binding induces the association of T*β*RI, which allows T*β*RII to phosphorylate serine residues in the GS domain of the type I receptor. This activates the kinase domain of the type I receptor, which phosphorylates Smad effector proteins (Smad2 and Smad3). Once phosphorylated, Smad2 and Smad3 form a complex with Smad4 and translocate from the cytoplasm to the nucleus, where they specifically interact with other transcription factors, coactivators, or repressors such as AP-1. This regulates the transcription of TGF-*β* dependent genes, whose products are involved in biological activities including differentiation, growth control, apoptosis, and ECM synthesis [3, 11, 43] (Figure 2).

The diversity of TGF-*β* signaling is determined not only by the existence of different ligands, receptors, mediators, or Smads that act in concert but also by TGF-*β*'s own ability to activate other signaling pathways [44]. TGF-*β* may indirectly play a role in apoptosis, in the mesenchymal-epithelial transition, migration, proliferation, differentiation, and extracellular matrix formation via the activation of other non-Smad or Smad-independent signaling pathways such as TAK1 (TGF1 associated kinase), Erk (extracellular signal-regulated kinases), p38, mitogen-activated protein kinase (MAPK), PI3K-Akt, and JNK (terminal N kinase) [43]. For example, the ERK-MAPK pathway is activated by different growth factors including oncogenic Ras and TGF-*β* that are frequently upregulated in cancer. TGF-*β*-induced Ras signaling promotes the epithelial-mesenchymal transition (EMT) [45]. Moreover, TGF-*β* activates the PI3K-Akt signaling pathway. Although the molecular mechanisms are not yet fully understood, T*β*RII and T*β*RI seem to be necessary for activation of the PI3K-Akt signaling pathway. Because the PI3K-Akt pathway plays a crucial role in tumor progression, the identification of the mechanisms by which TGF-*β* can modulate these pathways may provide new therapeutic targets in cancer treatment [43, 46].

## 6. TGF-*β* Functions

The multifunctionality of TGF-*β* family members has been related to several disease entities and cellular processes, including the development of fibrosis and malignant tumors. The great variety of specific responses to TGF-*β* is mostly dependent on the TGF-*β*1 isoform, which is the most broadly studied and most frequently detected member of the family. TGF-*β* may act as an indirect mitogen on mesenchymal cells or as a stimulator of extracellular matrix protein synthesis. TGF-*β* is also a potent inhibitor of the proliferation of epithelial, endothelial, lymphoid, and myeloid cells. Thus, TGF-*β* can be considered the prototype of a multifunctional cytokine due to the diverse effects it has on different target cells [3, 47].

Studies focused on establishing the role of TGF-*β* suggest that it plays a role in immune and inflammatory processes because it suppresses the growth and differentiation of many immune cell lineages, including T and B cells [48]. Aside from regulating the proliferation of immune system cells, TGF-*β* also regulates the expression of adhesion molecules, particularly in the bone marrow and in the thymic microenvironment. TGF-*β* also acts as a fibroblast, monocyte, and neutrophil chemoattractant and inhibits immune system activation mediated by antigen or interleukins (IL) [49].

## 7. TGF-*β* and Allergic Disease

TGF-*β* plays a key role in asthma because it mediates leukocyte chemotaxis to pulmonary tissue, a crucial step in the genesis and maintenance of an inflammatory response [50]. TGF-*β* also acts as a fibrogenic and immunomodulatory factor, thus playing a pivotal role in airway structural changes in asthma patients. Asthma is defined as a chronic inflammatory airway disease that leads to various degrees of airway inflammation and affects millions of individuals worldwide. Airway inflammation in asthma is mediated by Th2 cytokines, so the involved cells are basophils, eosinophils, and mastocytes [51]. The cytokines IL-4, IL-5, and IL-13 play a prominent role in asthma's inflammatory cascade [52].

TGF-*β* is a key molecule in the repair of the airway epithelium not only in allergic diseases such as asthma and allergic rhinitis but also in fibrosis and infectious disease [50].

TGF-*β*1 is a pleiotropic and multifunctional growth factor with an important immunomodulatory role and fibrogenic properties. TGF-*β* is a master regulator of the immune response and exerts important anti-inflammatory functions. On the other hand, it has chemoattractant properties and leads to the rapid accumulation of macrophages, granulocytes, and other cells at the site of inflammation [53]. TGF-*β*1 can also induce TH17 cell differentiation, producing cells that are capable of producing large quantities of IL-17 and perpetuating an acute inflammatory process. TGF-*β* also fosters the secretion of other inflammatory cytokines and recruits granulocytes that amplify the immune response [54]. Moreover, TGF-*β*1 has anti-inflammatory and immunosuppressive properties, as reflected by the inhibition of immune cell differentiation (Th1 and Th2 cells and B cells) and cytokine production (IFN-*γ* and IL-2). Finally, TGF-*β* is critical to the development and differentiation of regulatory T cells (T_reg_) [55].

### 7.1. TGF-*β* and Tissue Repair (Fibrosis)

The development of cell and molecular biology tools has allowed a better understanding of the processes involved in tissue repair. Tissue repair implies a coordinated sequence of biological events that begin with platelet-mediated hemostasis. Next, inflammatory cells including fibroblasts enter the wound site and begin the formation of new extracellular matrix and blood vessels (granulation). Finally, cells proliferate to reconstitute the damaged tissue. TGF-*β* plays a very important role in the coordination of these events [50].

TGF-*β* is a powerful stimulus for fibroblast proliferation and ECM deposition [56]. The extracellular matrix is a dynamic superstructure composed of self-aggregating molecules including fibronectin, collagen, and proteoglycans, to which cells adhere via surface receptors known as integrins. TGF-*β*1 promotes the deposition of extracellular matrix and simultaneously stimulates cells to increase their production of matrix components while decreasing the release of proteases that can degrade ECM structure [57]. The sustained production of TGF-*β*, as a result of either tissue injury, a defect in TGF-*β* regulation, or both, leads to an imbalanced deposition of the ECM that underlies tissue fibrosis in chronic asthma [58].

Allergic asthma is characterized by Th2 chronic inflammation that leads to airway remodeling [8] and symptoms of decreased lung function. Airway remodeling refers to modifications to the normal structure of the airway wall and implies changes in its cellular and molecular composition and organization [24]. Airway remodeling is the result of airway repair after persistent inflammation. Airway remodeling implies a complex series of events including epithelial injury, hyperplasia of goblet cells, subepithelial fibrosis, smooth muscle cell hypertrophy and hyperplasia (ASMC), and vascular remodeling; each remodeling component contributes to pulmonary dysfunction [24, 58]. During the development of the asthmatic state, there are inflammatory cell infiltration and cytokine secretion, including TGF-*β*1, which regulates the airway remodeling process. Some authors have suggested that high TGF-*β* levels in the airways correlate with asthma severity [59, 60].

The three TGF-*β* isoforms (*β*1, *β*2, and *β*3) are expressed in the bronchial epithelium. TGF-*β*1 and TGF-*β*3 are expressed by eosinophils, lymphocytes, and macrophages. TGF-*β*1 is expressed in the vascular endothelium, smooth muscle cells, and fibroblasts and is bound to the subepithelial ECM [53, 61]. In healthy airways, TGF-*β* expression is limited to the airway epithelium, although mesenchymal cells, including fibroblasts and airway smooth muscle (ASM) cells, also express it in significant amounts [62].

During the development of fibrosis, TGF-*β*1 induces the expression of target genes including connective tissue growth factor (CTGF), smooth muscle *α*-actin (*α*-SMA), collagen, and plasminogen activator inhibitor (PAI). These target genes induce fibroblast chemoattraction, proliferation, and differentiation into myofibroblasts that synthesize ECM proteins such as fibronectin and collagen that, in turn, lead to ECM contraction. Fibroblasts play a crucial role in the regulation of fibrosis and in the pulmonary immune response after TGF-*β*1 activation [63]. TGF-*β*1 has been identified as the master switch in the induction of the epithelial-mesenchymal transition (EMT) that leads to the differential conversion of epithelial cells into fibroblasts and myofibroblasts. For this reason, the ECM has been proposed to be responsible for increases in fibroblasts and myofibroblasts in mucosa and for the collagen overproduction and fibrosis observed in asthma and other lung diseases such as chronic obstructive pulmonary disease (COPD) [53]. TGF-*β* expression increases in the airways of asthmatic patients due to both structural and inflammatory cell infiltrates. Eosinophils constitute between 70 and 80% of all cells expressing TGF-*β*1 in these patients' airways. Eosinophils are considered asthma markers due to their pivotal role in inflammation and remodeling [61, 64]. Interestingly, a correlation between increased TGF-*β*1 expression and increased eosinophils [65, 66] and macrophages [67] has been established. Likewise, an increase in TGF-*β*2 expression in asthmatic epithelium [68] has also been shown, and this increase correlates with an increase in the number of eosinophils and neutrophils after allergen challenge in patients with severe and mild asthma [59, 60, 69, 70]. There is scant information on TGF-*β*3 expression, although the available evidence suggests that there is no difference in TGF-*β*3 expression between controls and asthmatic patients [59, 69].

## 8. TGF-*β* in Cancer

TGF-*β* plays a tumorigenic role and can also act as a tumor suppressor depending on the cellular context and the tumor's stage [14]. TGF-*β* acts as an antitumor agent in early or primary cancer stages. However, in more advanced cancers, TGF-*β* favors tumor development. Cancer cells often avoid the growth inhibitory effects of TGF-*β* but maintain their responsiveness to TGF-*β*, thus promoting tumor progression [71].

Most cancers initially express a functional form of TGF-*β* as well as the proteins involved in the TGF-*β* signaling pathway. However, during transformation, tumor cells become resistant to the inhibitory effects of TGF-*β*, thus leading to cell proliferation, invasiveness, and an increase in metastatic potential. Abnormalities in TGF-*β* signaling have been shown to contribute to cellular proliferation, cancer development, and metastasis. This is favored by the inactivation of various components of the TGF-*β* receptor signaling system, such as T*β*RII and T*β*RI, or by Smad protein mutations that decrease the sensitivity to TGF-*β*'s inhibitory effects. An increase in TGF-*β* mediated promotion of the epithelial-mesenchymal transition (EMT) enhances invasiveness, metastases development, angiogenesis, and immune suppression. Enhanced invasiveness is observed when TGF-*β* is overexpressed in tumors including breast, colon, esophageal, gastric, liver, lung, kidney, pancreas, prostate, and brain cancers, malignant melanoma, and certain hematological diseases [72].

## 9. TGF-*β* as a Suppressor of Cancer Development

The most critical cellular effect of TGF-*β* is the suppression of proliferation. Its growth inhibiting properties have been demonstrated in normal epithelial cells and early tumor cells. However, growth inhibition hinges on the ability of TGF-*β* to suppress the expression and function of the c-Myc protooncogene or cyclin-dependent kinases (CDK) and even of CDK inhibition regulators such as p15, p21, and p27 [72, 73].

### 9.1. Cytostatic Effect

TGF-*β* plays a key role in the control of growth by means of its cytostatic effect and its effects on apoptosis. As a result of these functions, TGF-*β* is considered a tumor suppressor. Cancer cells have been shown to elude the TGF-*β* mediated antiproliferation effect by somatic mutation of some TGF-*β* signaling components or by interrupting TGF-*β*'s cytostatic effect [74].

Cell responses to TGF-*β* depend on the cell type and the associated physiological conditions. TGF-*β* stimulates different types of mesenchymal cells, including fibroblasts, but is a potent inhibitor of epithelial, endothelial, neural, and hematopoietic cells, including cells participating in the immune response [22]. The pivotal function of TGF-*β* is inhibition of cell cycle progression by controlling regulatory proteins involved in the process. Thus, TGF-*β* suppresses the function and expression of c-Myc via Smad3 [75–77].

**Figure 3:**
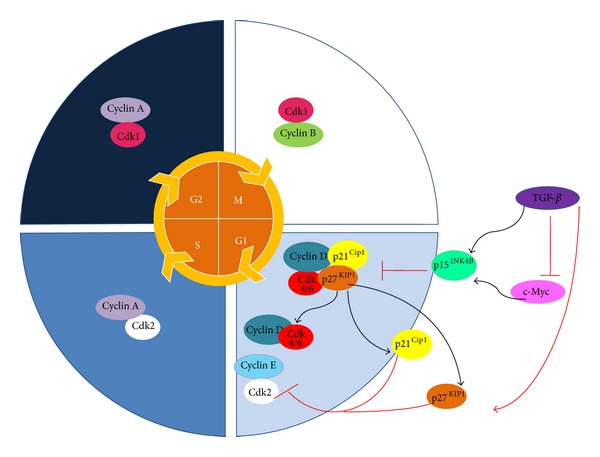
The role of TGF-*β* in cell cycle regulation. Physiologically, TGF-*β* is a potent cell cycle inhibitor, inducing the expression of p15^INK4B^ and suppressing c-Myc expression. p15^INK4B^ prevents the formation of cyclin D-CDK4/6 complexes and displaces p21^Cip1^ and p27^KIP1^ from the cyclin D-CDK4/6 complex. The inhibitors CIP/KIP can then inactivate other G1 to S phase complexes and inhibit the cell cycle. Low c-Myc levels allow TGF-*β* to induce the transcription of p15^INK4B^ and p21^Cip21^.

The cytostatic effect of TGF-*β* may also depend on the Rb protein because TGF-*β* maintains Rb in a state of hyperphosphorylation during early G1 phase [78]. TGF-*β* induces and potentiates the expression of the CDK inhibitors p15^INK4B^, p21^Cip1^, and p27^KIP1^ [52, 74]. The mechanism by which TGF-*β* regulates the cell cycle involves the induction of CKI p27^Kip1^ expression. p27^Kip1^ binds and inhibits cyclin-CDK2 complexes [73] and induces transcription of p15^INK4B^ [79] and p21^Cip1^ [80, 81]. Studies conducted on epithelial cells have shown that an increase in p15^INK4B^ leads to the binding of CDK4 and CDK6, thus displacing the p27^Kip1^ and p21^Cip1^ kinases that bind to and inhibit CDK2. A concomitant increase in p21^Cip1^ ensures a maximum blockade of CDK2 activity and cell cycle arrest in the G1-S transition phase [81] (Figure 3).

### 9.2. Proapoptotic Effect

Aside from the TGF-*β* mediated cytostatic properties that arrest the cell cycle, TGF-*β* also has a proapoptotic effect. However, TGF-*β* mediated apoptosis activation varies depending on the tissue and cell type [82].

The microenvironment influences how cells respond to apoptotic signals and to cell-cell or exogenous environmental signals. Apoptosis and dysregulation of proliferation are key factors in carcinogenesis because when cells no longer respond to apoptosis signals when DNA is damaged, tissues are more prone to tumorigenesis due to the accumulation of genetic mutations that allow them to proliferate autonomously [82]. Smad signaling regulates the expression of various genes in the apoptotic process, in a manner similar to the previously described cytostatic process. The critical genes regulated by Smad include the GADD45*β*r signaling factor domain, the Bcl-2 Bim homologous factor, the death-associated protein kinase (DAPK), the SHIP phospholipid phosphatase, and the TGF-*β*-inducible early response gene 1 (TIEG1) [83].

TIEG1 is a transcription factor that regulates the expression of other proapoptotic genes. Although its specific transcription mechanisms have yet to be described [83], TIEG1 appears to activate the mitogen-activated protein kinase kinase 4 (MKK4) signaling pathway, which in turn activates the p38 MAPK pathway, which leads to caspase-8 activation [84, 85]. TGF-*β* induces the expression and activation of the Fas receptor via Smad3; this leads to the activation of caspase-8 and apoptosis of gastric cancer and lymphoma [86, 87].

## 10. TGF-*β* as a Promoter of Tumor Growth

TGF-*β* may induce the development of metastases by increasing epithelial cell motility. This phenomenon involves the transition of epithelial into mesenchymal cells (EMT) that may be mediated by a TGF-*β*-induced increase in cellular plasticity. Tumor cells may use this pathway to withdraw from the primary tumor site via the circulation, thus allowing tumor cells to bud off the primary tumor and generate metastatic lesions at a secondary site [88].

The epithelial-mesenchymal transition (EMT) is a normal physiological process that is essential for embryonic development and tissue remodeling and repair. The essential element of EMT is a decrease in cell-to-cell adherence as a result of changes in cell adhesion molecules and the production of an extracellular matrix that allows the separation of cells from one another, as well as the loss of typical epithelial cell polarity [89]. Inappropriate reactivation of the EMT is commonly observed in human malignant tumors [90, 91].

Biochemically, EMT is characterized by a downregulation of E-cadherin expression and increased vimentin [92], ZO-1, vinculin, and keratin expression [5]. EMT may occur via a combination of Smad-dependent and Smad-independent signals [93]. The noncanonical signaling pathway may play an important role in EMT and includes Ras/MAPK, PI3K, Akt, Rho/ROCK, and NF-*κ*Bv [5, 45, 93, 94] as well as other factors such as *α*v*β*3 and Src *β*3 integrins that physically interact with T*β*RII and promote Src-mediated T*β*RI phosphorylation [82, 95].

### 10.1. TGF-*β* Promotes Tumor Angiogenesis

The formation of new blood vessels is necessary for malignant cells to obtain nutrients and oxygen for growth. Well-vascularized tumors tend to extravasate more easily into the systemic circulation, promoting the progression of tumors into metastases. Without vascularization, the tumor is unable to grow and, therefore, undergoes necrosis and has a more benign phenotype [96]. Thus, the formation and preservation of blood vessels are crucial for tumor progression and are important steps that could be blocked in cancer therapy [82].

Two main types of cells are required for blood vessel formation: endothelial cells (EC) and perimural cells. There are several distinct steps in angiogenesis [97, 98]. First, angiogenic factors such as vascular endothelial growth factor (VEGF) are induced and activate an angiogenesis cascade. Matrix metalloproteinases (MMPs) are then activated and promote extracellular matrix (ECM) degradation. Next, endothelial cells are released from the capillary wall and migrate to the site of angiogenesis [82]. TGF-*β* is capable of inducing angiogenesis by direct and indirect mechanisms. Directly, TGF-*β* induces the expression of vascular endothelial growth factor (VEGF) [99], and indirectly TGF-*β* release stimulates an increase in the expression of MMP-2 and MMP-9 by macrophages and monocytes infiltrating the tumor. This promotes a protein-rich microenvironment that facilitates migration and the invasive properties of the activated endothelial cells [86, 100]. Moreover, it has also been reported that endoglin, a transmembrane glycoprotein overexpressed by vascular endothelial cells during proliferation, acts as a coreceptor and interacts with TGF-*β* [101], protecting endothelial cells from the inhibitory properties of TGF-*β*. Endoglin activates ALK-1, which phosphorylates the alternative Smads (Smad1, Smad5, and Smad8) that promote endothelial cell proliferation, migration, and the transcription of other proangiogenic genes [102]. Thus, VEGF is implicated in tumor vascularization [98, 99, 103] and secondarily affects angiogenesis by acting as a monocyte chemoattractant protein that liberates other angiogenic cytokines [100]. TGF-*β* induction in angiogenesis correlates with cancer progression, but TGF-*β* can regulate angiogenesis at different levels during development and in carcinogenesis [82].

### 10.2. Mutations in TGF-*β* Signaling Components

Malignant cells can become resistant to the suppressor effects of TGF-*β* as a result of mutations and/or TGF-*β* receptor functional inactivation or due to abnormalities in the Smad signaling pathway. TGF-*β* acts as a tumor promoter and is often overexpressed in various types of cancer. Its plasma levels are increased in hepatocellular carcinoma, colon cancer, and prostate, lung, and breast cancer and high plasma TGF-*β* levels seem to correlate with an unfavorable prognosis [101].

Mutations in the genes encoding TGF-*β* signaling components have been detected in many tumors. These mutations can cause small variations in the structure of TGF-*β* or complete loss of its expression. Alterations in TGF-*β* signaling can potentially result in cancer development and progression. Mutations in T*β*RI, T*β*RII, Smad2, and Smad4 are common. In particular, T*β*RII inactivation leads to tumor dissemination and metastasis in a variety of carcinomas including colon [104], breast [105], pancreas [106], and intestinal neoplasias [106, 107].

## 11. Allergooncology

The debate about the relationship between allergy and cancer is not recent [108, 109]. The increasing prevalence of allergic disease in recent decades [110–112] and new discoveries about the immunology of cancer have increased the interest in this relationship [113, 114]. This association has been demonstrated primarily by epidemiological observations. Numerous epidemiological studies have investigated the association between a history of allergies and the risk of different neoplasias, such as pancreatic cancer, glioma, and childhood leukemia. There has been an expansion of the epidemiological literature aimed at investigating correlations between a clinical history of allergies and cancer development. Biological markers of a history of allergies have been evaluated, including those pertaining to immune function such as immunoglobulin E (IgE) levels [115]. However, the epidemiological data linking allergy and cancer development have yet to be confirmed in experimental models. The possible relationship between the two phenomena could contribute to the prevention of cancer.

To explain positive or direct associations, McWhorter proposed a “theory of antigenic stimulation” in 1988 [115–117], a theory that has since been revisited [118, 119]. The theory of antigenic stimulation suggests that allergies lead to chronic inflammation and cell growth stimulation, which increases the probability of mutations in actively dividing cells and the generation of aberrant clones. The relationship between chronic inflammation and cancer has been established [120]. According to the antigenic stimulation hypothesis, allergy symptoms directly increase the risk of developing cancer in any tissue or organ [121].

Likewise, two other hypotheses have attempted to explain the inverse relation between cancer and a history of allergies. The first is the “immunovigilance hypothesis” proposed by Burnet in 1957 [122] and subsequently revisited [123, 124]. The immunovigilance hypothesis states that individuals with efficient and robust immune systems effectively prevent cancer development by detecting and eradicating premalignant autologous cells before tumors develop.

However, this heightened immune response results in an exacerbated response to allergens such as pollen, mold, helminthes, and other extraneous particles. According to this hypothesis, allergy symptoms are secondary effects of hyperimmunity and effective immune vigilance. Thus, allergy symptoms* per se* do not directly promote cancer development, and an inverse correlation between allergies and cancer suggests a noncausal relationship [121, 124].

A second explanation of the inverse relation between cancer and allergy is “the prophylaxis hypothesis” [105, 124] that follows a Darwinian perspective. The prophylaxis hypothesis suggests that allergy symptoms develop due to natural selection and serve a useful purpose by removing toxins, pathogens, or extraneous particles from the body. Similarly, foreign substances or mutagens that could adhere and initiate the carcinogenic process are also removed. Allergy symptoms may serve as warning signals that “caution” the individual about environmental substances that are best avoided. According to the prophylaxis hypothesis, allergy symptoms may directly decrease the occurrence of cancer by rapidly clearing tissues of potential mutagenic toxins, microorganisms, and environmental pollutants and also by fostering the avoidance of these antigens in the future. Therefore, the inverse relation between allergy and cancer may be causal and not just a correlation.

In view of these contradictory theories, a new area of research has recently developed, bringing together the two disease entities: “allergooncology.” Allergooncology focuses on determining the interrelationships between cancer and the Th2 immune response. Recent data have advanced clinical observations to the point that we are beginning to understand the molecular mechanisms involved and the possible therapeutic approaches [125]. Epidemiological studies have suggested inverse associations between allergic disease and various malignant tumors, leading to the suggestion that immunoglobulin E (IgE) may destroy tumor cells. Several experimental strategies have been designed to evaluate IgE directed against specific tumor markers. IgE antibodies are elevated in the serum, in concentrations above those of other immunoglobulins. IgE displays a greater ability to induce antibody-dependent cellular cytotoxicity (ADCC) and phagocytosis reactions (ADCP). Nonspecific IgE binding to tumor cells is also a potent inducer of tumor-specific immunological memory. Th2 immunity may allow the administration of oral vaccines based on mimotopes, imitations of tumor antigens. Therefore, IgE antibodies not only play a role in antitumor vigilance but also may help to control a tumor when used for active or passive immunotherapy. IgE can bind to eosinophils, mastocytes, and macrophages, turning them into potent antitumor cells [102, 116, 117].

Interestingly TGF-*β* plays an important role in asthma because it mediates chemotaxis of leukocytes to lung tissue, which represents a key step in the genesis and maintenance of the inflammatory response. TGF-*β*1 also acts as an immunomodulator and a fibrogenic factor. Therefore, TGF-*β* plays an important role in the structural changes in the airways of patients with asthma and is closely associated with the severity of the disease [26, 56].

On the other hand, TGF-*β* plays different roles in other diseases such as cancer, where paradoxically TGF-*β* can both suppress and promote tumor progression. Suppressor activity is reflected in its antiproliferative and proapoptotic effects, but during tumor progression TGF-*β* promotes tumor growth following the acquisition of mutations in proteins that participate in the TGF-*β* signaling pathway. By inhibiting the antiproliferative properties of TGF-*β*, the tumor can use it to induce tumor motility, the epithelial-mesenchymal transition, and invasion and the development of metastases [22, 71, 78, 92].

However, there is evidence to suggest that overexpression TGF-*β*, its associated receptors (T*β*RI, T*β*RII), and the proteins involved in TGF-*β* signaling increase the survival of breast cancer patients [126]. A major study revealed that an allergic response to ovalbumin in sensitized mice protected the mice against Ehrlich tumor growth in the mouse footpad. Significantly decreased tumor growth was observed due to an increase in apoptosis in allergic mice. These results suggest that a concomitant allergic condition could decrease tumor progression through increased apoptosis of tumor cells [127]. Indeed, these findings suggest a possible mechanism for the reduction of cancer incidence observed in allergic individuals.

The importance of TGF-*β* has also been reported in a mouse model of allergic lung inflammation and lung cancer induced by urethane. Here, inhibiting TGF-*β* signaling in Clara cells of the lung by means of Smad7 overexpression, which in turn inhibited the activity of TGF-*β*, increased tumor lesions [128]. These results are evidence that TGF-*β* may control the development of neoplasia and suggest a possible interaction between allergic diseases and cancer. These findings will guide allergooncology, whose purpose is to understand and decipher the cellular and molecular mechanisms involved in scenarios where two different pathological entities can coexist.

Very recently, Porretti et al. [129] demonstrated that conditioned media (CM) from normal fibroblasts differentially strengthen the EMT process in breast cancer cells with different malignant behavior. Remarkably, histamine prevented the activation of fibroblasts and the EMT related changes induced in tumor cells by fibroblast CM. It is well documented that activated fibroblasts secrete a variety of soluble growth factors and chemokines (TGF-*β* among others) that have a paracrine effect on different cell types, driving tumor growth and progression [130, 131]. In this regard, histamine may be modulating the secretion of a soluble factor from fibroblasts. Further studies need to be conducted to identify the factor.

Histamine-storing cells such us mast cells, basophils, macrophages, and histaminergic neurons produce high concentrations of histamine. In adenocarcinomas such as breast cancers, infiltrating mast cells and macrophages are abundant. These cells accumulate in the stroma in response to numerous chemoattractants and they play a dual role in tumor biology by secreting factors that may induce tumor cell growth or death, angiogenesis, matrix remodeling, and immunosuppression [132, 133]. Histamine is one of such factors. Endogenous histamine may also influence fibroblast-tumor cell interactions [129]. During the last decade, histamine has been used as an immunomodulator in phases II and III clinical trials for melanoma, metastatic renal cell carcinoma, and acute myeloid leukemia. Histamine is well tolerated and shows no side effects [134–137]. Therefore, the inhibitory effects of histamine on tumor cell proliferation [138, 139] strongly suggest that histamine is a potential agent that should be considered in the investigation for new combined treatments. These observations support the idea that, in allergic diseases, histamine may play a very important role in cancer progression.

This review has attempted to present a new view on the possible association of a common molecule, TGF-*β*, with two different conditions: allergic disease and cancer. However, the exact mechanisms involved in allergic disease and cancer need to be studied further to develop strategies that provide new and better therapeutic targets in situations where both diseases coexist.

## Abbreviations

ActR-II: Type II activin receptor
ActR-IIB: Type IIB activin receptor
Acts: Activins
ALK: Activin-like kinase receptor
AMHR: Anti-Müllerian hormone receptor
ARTS: Apoptosis-related protein in the TGF-*β* signaling pathway
ASM: Airway smooth muscle cells
ASMC: Bronchial smooth muscle cells
BAL: Bronchoalveolar lavage
BMP: Bone morphogenic proteins
BMPR-II: BMP type II receptor
CDK: Cyclin-dependent kinases
Co-Smads: Common Smad protein
CTGF: Connective tissue growth factor
DAPK: Death-associated protein kinase
DAXX: Fas-associated receptor
EC: Endothelial cells
EGF: Epidermal growth factor
ELF: Embryonic liver fodrin
EMT: Epithelial-mesenchymal transition
EndMTs: Endothelial-mesenchymal transitions
COPD: Chronic obstructive pulmonary disease
ECM: Extracellular matrix
Erk: Extracellular signal-regulated kinase
Fgf1: Fibroblast growth factor 1
FGF10: Keratinocyte growth factor 2
FGF7: Keratinocyte growth factor 7
FTLP: Ferritin light polypeptide pseudogene
GDFs: Growth differentiation factors
GS: Glycine-serine rich domain
G-TSF: Glioblastoma-derived T cell suppressor factor
HCC: Hepatocellular carcinoma
Hgf: Hepatocyte growth factor
Hgs: HGF-regulated tyrosine kinase substrate
HNSCC: Neck carcinoma cells
IFN-*γ*: Gamma interferon
IgE: Immunoglobulin E
Igf1: Insulin-like growth factor type 1
Igf2: Insulin-like growth factor type 2
IgG: Immunoglobulin G
IL: Interleukin
Inhs: Inhibins
I-Smads: Inhibitory Smad protein
JNK: c-Jun N-terminal kinase
LAP: Latency-associated proteins
LLC: Large latent complex
LTBP: Latent TGF-*β* binding protein
MAPK: Mitogen-activated protein kinase
MET: Mesenchymal-epithelial transition
MIS: Müllerian inhibiting substance
MKK4: Mitogen-activated protein kinase kinase 4
MMPs: Metalloproteinases
NK: Natural killer cells
PAI: Plasminogen activator inhibitor
PI3K: Phosphoinositol 3 kinase
pRb: Retinoblastoma protein
ROCK: Rho-associated protein kinase
R-Smads: Receptor-activated Smad proteins
SANE: Smad antagonist effector 1
SARA: Smad anchor for receptor activation
SHIP: Phospholipid phosphatase
SLP: Small latent complex
TGF-*β*: Transforming growth factor *β*
TIEG1: TGF-*β*-inducible early response gene 1
TIMPs: Protease inhibitors
TRAP 1: TGF-*β* receptor-associated protein 1
T_reg_: Regulatory T cells
TSP: Thrombospondin
T*β*RI: TGF-*β* receptor I
T*β*RII: TGF-*β* receptor II
VEGF: Endothelial and vascular growth factor
ZO-1: Tight junction protein
*α*-SMA: Smooth muscle *α*-actin.
